# Facteurs de stress en entreprise: cas de 223 salariés des entreprises privées formelles (EPF) de la ville de Ouagadougou

**DOI:** 10.11604/pamj.2021.40.50.19670

**Published:** 2021-09-21

**Authors:** Brigitte Nana, Amidou Sawadogo, Ahmed Kaboré, Libérat Tanimomo

**Affiliations:** 1 Institut des Sciences du Sport et du Développement Humain (ISSDH), Université Joseph Ki-Zerbo, Ouagadougou, Burkina Faso,; 2 Institut National de la Jeunesse, de l´Education Physique et du Sport (INJEPS), Université d´Abomey Calavi, Abomey Calavi, Bénin

**Keywords:** Stress professionnel, entreprise, modèle de Karasek, modèle de Siegrist, Occupational stress, company, Karasek´s model, Siegrist´s model

## Abstract

**Introduction:**

au Burkina Faso, le stress professionnel constitue un problème de santé publique. L´objectif de cette étude était d´identifier les facteurs de stress chez les salariés des entreprises privées formelles de la ville de Ouagadougou.

**Méthodes:**

l´enquête a été réalisée au moyen des questionnaires de Karasek à 26 items et de Siegrist à 23 items. Les analyses ont été réalisées sur la base des modèles d´analyse validés de Karasek et de Siegrist et sur le logiciel SPSS.

**Résultats:**

nous avons enquêté 223 salariés (186 hommes et 37 femmes) âgés en moyenne de 36,70 ans ± δ = 33,25. En plus, 70,40% des salariés sont en job strain; 50,22% en iso strain et 52,02% en déséquilibre effort/récompense. Des analyses post hoc ont permis de mettre en évidence les facteurs de stress suivants: gros efforts fournis et faible pouvoir de décision.

**Conclusion:**

la présente étude a révélé l´existence du stress chez les salariés des entreprises et a permis de mettre en évidence l´importante nécessité de combiner les questionnaires de Karasek et de Siegrist dans l´étude des facteurs de stress.

## Abstract (en)

**Introduction:**

in Burkina Faso, work-related stress is a public health problem. The purpose of this study is to evaluate factors of stress among formal private sector employees in the city of Ouagadougou.

**Methods:**

we conducted a survey using the 26-item scale derived from Karasek and the 23-item scale derived from Siegrist. Validated Karasek and Siegrist´s models as well as SPSS software were used to analyze data.

**Results:**

we surveyed 223 employees (186 men and 37 women) with an average age of 36.70 years ± δ = 33.25. In addition, 70,40% of employees had job strain; 50,22% iso strain and 52,02% effort-reward imbalance. Post-hoc analyses showed the following stress factors: great efforts and poor decision-making ability.

**Conclusion:**

this study confirms the presence of stress among private sector employees and highlights the importance of combining Karasek and Siegrist’s questionnaires in the study of stress factors.

## Introduction

La problématique des risques psychosociaux au travail est devenue de nos jours une question sociale et politique car elle retient l´intérêt de tous et soulève une émotion intense lorsqu´elle se présente sous la forme d´événements dramatiques [1]. Dans le domaine du travail, le stress professionnel constitue le principal risque psychosocial chez les salariés [2]. Véritable risque pour la santé physique et mentale chez les travailleurs, son ampleur varie selon les différents types de professions exercées [3]. Le stress serait à l´origine de 50% à 60% de l´ensemble des journées de travail perdues et coûterait 20 milliards d´euros chaque année à l´Europe et aux Etats-Unis [4].

Défini comme un phénomène qui survient lorsqu´il y a un déséquilibre entre la perception qu´une personne a des contraintes que lui impose son environnement et la perception qu´elle a de ses propres ressources pour y faire face [5], le stress professionnel a fait l´objet de plusieurs conceptualisations dont les plus utilisées dans la littérature sont celle de Karasek et celle de Siegrist. Le modèle de Karasek définit le stress comme un état de tension dû à ce qui est exigé de la personne et des ressources dont cette personne dispose tandis que celui de Siegrist le considère comme une tension émanant du déséquilibre entre les efforts élevés et des récompenses faibles.

Sur la base de ces modèles d´analyses, nombreux sont les auteurs qui ont décrit les facteurs de stress. En 2012, [6] Siegrist a observé que l´expérience stressante au travail est vécue quand il y a un manque de réciprocité entre les efforts fournis et les récompenses reçues en retour en terme d´argent, de promotion, de sécurité du travail et d´estime. C´est ainsi qu´il a affirmé que les conditions de travail et le management de l´emploi contribuent au développement significatif des maladies cardio-vasculaires en particulier dans les sociétés modernes où les demandes psychologiques et les menaces deviennent répandues. De même, il a été rapporté que les hautes exigences de travail et le faible contrôle étaient les facteurs de stress en entreprise [7]. Sur une population d´employés d´industrie en métallurgie en Finlande, les facteurs de stress suivants ont été rapportés [8]: 1) selon le modèle du job strain: une haute demande psychologique au travail et un bas contrôle; 2) selon le modèle équilibre effort-récompense: un bas salaire, un manque d'approbation sociale et peu d'occasions de promotion de carrière en relation avec les efforts exigés au travail.

En 2001, des auteurs ont conclu que l´environnement psychosocial au travail résume les contraintes psychologiques, sociales et relationnelles liées à l´organisation du travail [9]. Selon ces auteurs, les instruments de Karasek et de Siegrist ont permis de dégager une association de type causal entre l´exposition aux facteurs psychosociaux au travail et divers aspects de la santé. De plus, chez les employés de Banque à Quatari, des facteurs de stress tels que le roulement vague donné aux employés et les charges de travail ont été rapportés [10]. De même chez les salariés en entreprise en Inde, il est ressorti dans une étude que les facteurs de stress concernent toutes les catégories professionnelles [11]. En utilisant le questionnaire de Siegrist, un auteur [12] a identifié cinq facteurs de stress: les efforts fournis, l'estime de soi, les récompenses, la sécurité du travail et le sur-engagement. En analysant l´impact du stress professionnel sur la performance, des études ont permis d´identifier trois principales causes du stress: la charge de travail, les conflits de rôle et les faibles récompenses inadéquates [13].

En définitive, bien que les facteurs individuels et personnels interviennent dans l´équation du stress, les causes du stress doivent être recherchées dans l´organisation du travail, l´environnement de travail et la structure organisationnelle de l´entreprise [14]. Sur la base du « Job content questionnaire » de Karasek, des auteurs ont rapporté les facteurs de stress suivants: une forte demande psychologique combinée à une faible latitude décisionnelle (industries agro-alimentaire, automobile) [15].

S´il existe une abondante littérature sur le stress professionnel dans les pays du nord, il y a cependant moins d´écrits sur le stress dans les pays du sud. C´est ainsi que ces auteurs [16] écrivaient à l´intention des employeurs et des représentants des travailleurs dans les pays en développement que le stress professionnel est un risque actuel. Il concerne tous les secteurs d´activité et toutes les catégories socio-professionnelles. En effet, dans les pays comme le Burkina Faso, les entreprises privées emploient une masse critique de la population active [17]. Moteur du développement économique, ces entreprises participent à hauteur de 20,2% du Produit Intérieur Brut [18]. En revanche, elles demeurent des lieux potentiellement générateurs de stress à cause de la privatisation et de la libéralisation du secteur par l´Etat [19,20]. Ces contraintes sont aggravées du fait de la non-disponibilité de l´emploi dans un contexte où le taux de chômage est estimé à plus de 6,6% de la population active [21]. Même si les différents écrits sur le stress en Afrique et particulièrement au Burkina Faso ne sont pas bien documentés, le constat sur l´existence du stress dans les entreprises est réel. L´objectif de cette étude est de mesurer le stress auquel les salariés des entreprises sont confrontés à travers ces facteurs associés.

## Méthodes

**Cadre de l´étude:** l´étude a été réalisée dans la ville de Ouagadougou chez les salariés des entreprises privées formelles.

**Type d´étude:** cette étude était de type transversal à visée descriptif et analytique. Elle a porté sur le stress professionnel chez les salariés des entreprises. Le stress professionnel est défini comme un déséquilibre ressenti entre les capacités du salarié perçues comme insuffisances et les contraintes de la situation.

**Echantillonnage/échantillon:** afin d´obtenir notre échantillon voulu, nous avons utilisé un échantillonnage aléatoire simple qui nous a permis de retenir cinq entreprises privées formelles sur l´ensemble des entreprises prisées formelles. Sur ces cinq entreprises, trois seulement ont donné leur autorisation d´enquête. La taille de l´échantillon a été déterminée par la formule de Schwartz [22] à travers laquelle on a retenu une taille de 223 salariés âgés en moyenne de 36,70 ans avec un écart type de 33,25 ans.

**Outils de collecte des données:** l´étude a été réalisée au moyen des questionnaires semi-structurés validés de Karasek à 26 items [23] et de Siegrist [24].

**Variables de l´étude**

***Variable dépendante:*** le stress vécu par les salariés dans les entreprises constitue la variable dépendante de cette étude. L´interprétation a été faite selon les recommandations suivantes: pour Karasek: job strain ou « tension au travail » est la combinaison d´une faible latitude décisionnelle et d´une forte demande psychologique. En pratique, si le score de la demande psychologique est supérieur à 20 et le score de la latitude décisionnelle est inférieur à 70, le salarié est dans le cadran « tendu » et est donc considéré en situation de « job strain » [22]. Quant à l´iso strain, il est la combinaison d'une situation de job strain et d'un faible soutien social inférieur à 24.

***Variables indépendantes:*** ce modèle admet trois variables indépendantes qui sont: la latitude décisionnelle, la demande psychologique et le soutien social. Les variables d´intérêts à ce niveau sont le job strain et l´iso strain. Pour Siegrist, un ratio > 1 définit les salariés exposés à un déséquilibre entre efforts et récompenses.

***Variables indépendantes:*** elles sont décrites à partir de ce modèle qui sont: les efforts et les récompenses. La variable d´intérêt constitue le déséquilibre entre les efforts et les récompenses.

**Gestion et analyse des données:** les calculs ont été effectués selon les modèles d´analyse de Karasek et Siegrist sus-cités (Karasek et Siegrist). Le traitement et l´analyse des données ont été réalisés sur le logiciel SPSS (version 21). Le test d´Anova à un facteur pour échantillon indépendant a été réalisé pour les comparaisons des scores dans les trois entreprises. Ensuite, le Test Poc Hoc de LSD a été effectué pour comparer les entreprises deux à deux en fonction du secteur d´activité.

En somme, les analyses ont consisté à: 1) construire les scores selon le modèle de Karasek par entreprise et pour l´échantillon d´étude; 2) calculer le ratio selon le modèle de Siegrist par entreprise et pour l´échantillon d´étude; 3) comparer les facteurs de stress de Karasek et de Siegrist en fonction du secteur d´activité.

**Considérations éthiques:** des structures compétentes en la matière ont été approchées et il a été suggéré de passer par l´Office de la Santé des Travailleurs (OST) qui nous a délivré une lettre de recommandation. Pour ce faire, nous avions eu recours à deux lettres de recommandations et des lettres d´autorisation d´enquête pour pouvoir s´entretenir avec les salariés.

## Résultats

### Description des variables de l´étude

**Figure 1:**
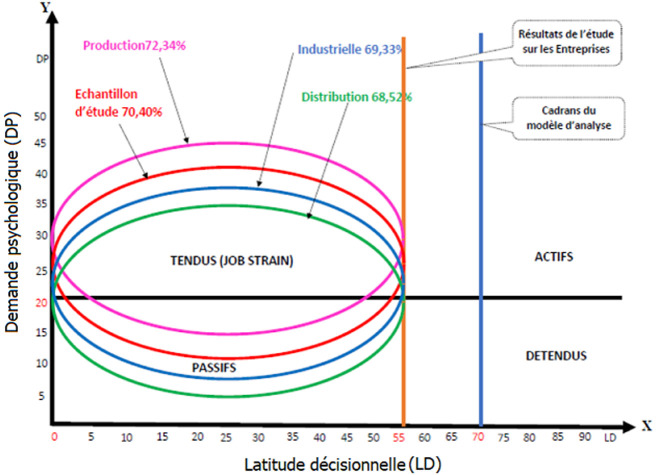
répartition des facteurs de stress des salariés des entreprises privées formelles (EPF)

**Figure 2:**
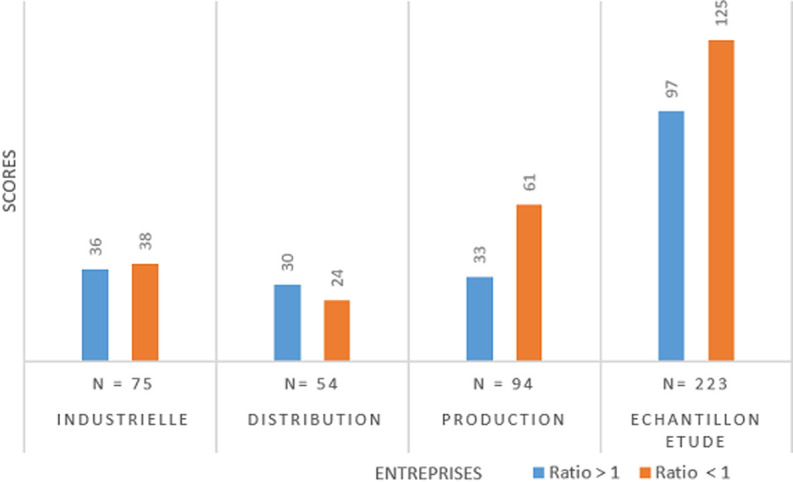
proportion des salariés ayant un ratio strictement supérieur à 1 en fonction du secteur d´activité

En s´inspirant des recommandations de Karasek, la Figure 1 présente les 4 cadrans du modèle d´analyse de cet auteur sur les facteurs de stress professionnel: les salariés tendus ou en situation de job strain; les salariés passifs d´une part et d´autre part les salariés actifs et les salariés détendus. En plaçant les scores calculés de cette étude sur ce cadran, il apparait que tous les salariés se retrouvent dans le cadran de job strain et des passifs; c´est ce qu´indiquent les courbes enchevêtrées. La Figure 2 présente les scores calculés en fonction du modèle d´analyse de Siegrist. La lecture de cette figure montre un ratio strictement supérieur à 1 pour la grande majorité des salariés des entreprises de production et pour l´échantillon d´étude. En effet, pour les salariés de l´entreprise de distribution et dans une certaine mesure pour l´entreprise industrielle, il y a plus de personnes en équilibre effort/récompenses que de personnes en déséquilibre (55 contre 39). Cette figure révèle donc que pour l´ensemble de l´échantillon, il y a un manque de réciprocité entre les efforts fournis et les récompenses. Par contre, pour l´entreprise industrielle, il y a une tendance de réciprocité entre les efforts et les récompenses.

### Comparaisons des différents scores de l´étude en fonction du secteur d´activité

Ce tableau indique la moyenne des scores de la latitude décisionnelle et des efforts en fonction du secteur d´activité. S´agissant de la latitude décisionnelle, le tableau montre que la différence entre les trois groupes de salariés est très significative (F = 4,32, ddl =222, avec p = 0,01). On peut dire que la latitude décisionnelle est différente selon le type d´entreprise avec un effet de taille moyenne de r = 0,20. Par ailleurs, les comparaisons post hoc des différents secteurs d´activité au niveau de la latitude décisionnelle (comparaison des entreprises deux à deux) ont donné pour le Test LSD, les résultats suivants: 1) la comparaison entre l´entreprise industrielle et l´entreprise de distribution donne un résultat significatif de p= 0,01; 2) la comparaison entre l´entreprise de distribution et l´entreprise industrielle a donné un résultat significatif de p= 0,01; 3) la comparaison entre l´entreprise de production et l´entreprise de distribution a donné un résultat très significatif de p = 0,00; 3) la comparaison entre l´entreprise de production et l´entreprise de distribution donne aussi un résultat très significatif de p = 0,00. De même pour les efforts, la différence entre les trois groupes de salariés est très significative (F = 19, 92 ddl = 222, p = 0,00). Cela montre que les efforts fournis par les salariés diffèrent selon le type d´entreprise. La taille d´effet est de 42%, ce qui signifie un effet de grande taille (r = 0,42).

Les comparaisons post hoc des différents secteurs d´activité au niveau des efforts (comparaison des groupes deux à deux) ont aussi donné pour le Test LSD, les résultats suivants: 1) la comparaison entre l´entreprise industrielle et l´entreprise de distribution donne un résultat significatif de p = 0,01; 2) la comparaison entre l´entreprise de distribution et l´entreprise industrielle donne un résultat significatif de p= 0,01; 3) la comparaison entre l´entreprise de distribution et l´entreprise industrielle donne un résultat très significatif de p = 0,00; 4) la comparaison entre l´entreprise de production et l´entreprise industrielle donne aussi un résultat très significatif de p = 0,00.

## Discussion

L´objectif de l´étude était d´identifier les facteurs de stress chez les salariés des entreprises privées formelles. Les résultats des différentes analyses ont montré que les facteurs de stress de notre échantillon s´inscrivent dans la conceptualisation de Karasek et de Siegrist. En effet, les déterminants du stress lié au travail chez les salariés des entreprises au Burkina Faso se résument à une faible latitude décisionnelle, une forte demande psychologique, un soutien social faible, un déséquilibre entre les efforts fournis et les récompenses selon les modèles d´analyse de Karasek et de Siegrist. Ces résultats traduisent le fait que les salariés de ces entreprises travaillent non seulement dans une situation de job strain et d´iso strain mais aussi dans une situation de travail nécessitant de grands efforts physique et mental sans récompenses conséquentes. Selon les modèles d´analyse de ces auteurs [25,26], ces résultats révèlent des charges de travail physiques et mentales importantes associées à un faible pouvoir de décision et une inadéquation entre l´effort fourni et les rémunérations: ce qui est à l´origine du stress vécu chez ces salariés.

En considérant les deux premiers axes de Karasek, la latitude décisionnelle et la demande psychologique, il est apparu que 70,40% de la population d´étude se trouve dans une situation de job strain contre 21,52% en situation de passivité (Figure 1). Le modèle de Karasek présente deux diagonales: un axe de tension ou de pression, qui s´échelonne du travail détendu au travail sous pression et un axe d´apprentissage allant du travail passif au travail actif [27]. La Figure 1 décrit la situation des salariés des entreprises sur ces diagonales. Il apparait un enchevêtrement des courbes au-dessus de la ligne médiane principalement dans la partie du travail tendue ou job strain se traduisant par une demande psychologique élevée et une latitude décisionnelle faible [22]. Parmi ces salariés en situation de job strain, une faible proportion se retrouve dans le cadran des passifs. La Figure 1 met en évidence l´existence d´une faible latitude décisionnelle (LD) (score < 70) située sur l´axe horizontal et d´une forte demande psychologique (DP) (score > 20) située sur l´axe vertical. Quant à la troisième dimension définie plus tard par Karasek et Theorell, le calcul des scores a révélé que 50,22% de cette même population se trouve en iso strain [28]. C´est dire que la grande majorité des salariés des entreprises travaille dans une situation de job strain et d´iso strain.

Cette description synoptique de la situation professionnelle des salariés des entreprises privées formelles au Burkina Faso corrobore celle décrite dans l´enquête nationale Sumer [22] chez les salariés français, qui a permis de dresser un état des lieux des expositions des dits salariés aux principaux risques professionnels en France métropolitaine [29]. En effet, depuis la réalisation de cette enquête, nombreux sont les auteurs qui utilisent ces résultats comme référence pour l´évaluation du stress professionnel [7-9, 15]. Pour ces auteurs, les hautes exigences de travail et le faible contrôle sont les principaux facteurs de stress en entreprise. En outre, les analyses ont rapporté que quel que soit le secteur d´activité (agro-alimentaire, transport ou hydrocarbure), ce sont les conditions de travail et l´environnement psychosocial qui génèrent la tension. C´est dans ce sens que qu´il a été précisé que les facteurs de stress de Karasek sont les mêmes dans tous les domaines [30]. C´est ainsi que des auteurs ont mis l´accent sur deux caractéristiques du travail: la demande et le contrôle [28]. Car pour eux, pour répondre aux diverses sollicitations imposées par le travail, les travailleurs ont besoin d´un certain degré de contrôle pour prévenir une éventuelle situation de tension.

Par ailleurs, la Figure 2 montre que 52,02% des salariés sont en déséquilibre entre efforts et récompenses. C´est dire que ces salariés consentissent de grands efforts pour la réalisation des tâches, mais ne sont pas conséquemment récompensés. Dans ses travaux de recherches en 2012, Siegrist [6] a observé que l´expérience stressante au travail est vécue quand il y a un manque de réciprocité entre les efforts fournis et les récompenses reçues en retour en terme d´argent, de promotion, de sécurité du travail et d´estime. C´est ainsi qu´il a été prouvé que la probabilité d´éprouver un stress élevé au travail était corrélée positivement avec le revenu [31]. Par exemple, l´auteur affirme que les salariés dont le revenu était égal ou supérieur à 40000 dollars avaient un niveau moins élevé de stress au travail comparativement à 28% de ceux ayant un revenu inférieur à 20000 dollars et à 42% de ceux dont le revenu variait entre 20000 dollars et 39.999 dollars. En revanche, il y a une faible tendance de réciprocité entre les efforts et les récompenses au niveau de l´entreprise industrielle. Cette tendance n´exclut pas l´inexistence du stress, car les facteurs de stress sont multiples et multiformes, en témoigne la Figure 1.

De nos jours, l´évaluation des facteurs de stress dans la littérature recommande la combinaison du modèle de Karasek et de Siegrist car l´association de ces deux modèles permet une prise en compte complète des facteurs de stress [7,9]. C´est ce qu´indique le Tableau 1. Les analyses croisées ont permis de mettre en évidence l´importante nécessité de combiner le questionnaire de Karasek et de Siegrist dans l´évaluation des facteurs de stress: p = 0,01 pour la latitude décisionnelle de Karasek et p = 0,00 pour les efforts extrinsèques de Siegrist. Ces résultats montrent que les facteurs de stress de l´échantillon d´étude se résument comme suit: gros effort fourni et faible pouvoir de décision. C´est dire donc que dans ces entreprises, les salariés subissent des contraintes physiques et mentales et n´ont aucune marge de manœuvre en matière de prise de décision. Ils y restent malgré tout car les offres d´emploi sont contingentées d´autant plus que le taux de chômage est élevé [21]. De plus, nombreux sont les auteurs qui ont montré que les plus importantes sources de stress dans l´organisation du travail sont la surcharge quantitative de travail et les récompenses inadéquates [4, 9, 13]. C´est en sens que certains auteurs ont souligné que les instruments de Karasek et de Siegrist permettent de dégager une association de type causal entre l´exposition aux facteurs psychosociaux au travail et divers aspects de la santé, en particulier les maladies cardio-vasculaires [9].

**Tableau 1:** comparaisons des facteurs de stress en fonction du secteur d´activité

Facteurs de stress	Entreprises	N	X	F	Valeur de P	<0,05 = *
Latitude décisionnelle	Industrielle	75	42,45	4,32	0,01	
	Distribution	54	40,30			
	Production	94	42,53			
Efforts	Industrielle	75	11,20	19,92	0,000	
	Distribution	54	9,96			
	Production	94	8, 85			

En somme, l´association des outils de Karasek et de Siegrist permet d´avoir une meilleure approche du vécu du stress chez les travailleurs [32]. Cela a été mis en évidence à travers des études d´envergure nationale notamment l´étude de Belgique Belstress [33] et de l´enquête Sumer en France [34].

En revanche, les secteurs d´activité ont montré une différence significative par rapport aux facteurs de stress. Que l´on soit salarié de l´entreprise industrielle, de production ou de distribution, le stress perçu est quasi similaire chez tous les salariés certes, mais les contraintes physiques et psychologiques diffèrent selon le secteur d´activité. C´est ce que révèlent les résultats des analyses post hoc. En effet, les analyses au niveau de la latitude décisionnelle de Karasek, des efforts extrinsèques de Siegrist se sont avérées significatives selon le type d´entreprise avec un effet de taille respectivement de r = 0,20 et de r = 0,42. Ces résultats mettent en exergue non seulement les deux principaux facteurs de stress qui sont: gros effort physique et faible pouvoir de décision; mais aussi indiquent que l´interaction entre l´individu et l´environnement psychosocial du travail est variable en fonction du secteur d´activité [35].

Somme toute, il ressort de nos analyses que le stress professionnel est vécu au quotidien chez les salariés dans les entreprises sus-citées. Cependant, ce stress est non exprimé du fait du contexte culturel burkinabé qui banalise la situation pourtant préoccupante. L´utilisation de ces modèles a permis non seulement de mesurer objectivement le stress chez ces salariés mais aussi de contribuer à fournir un cadre théorique sur lequel de futures recherches pourraient s´appuyer pour élaborer des recommandations pour l´amélioration de la qualité de vie au travail [25, 26].

## Conclusion

La présente étude avait pour but de mesurer les facteurs de stress des salariés des entreprises privées formelles dans la ville de Ouagadougou. Cette enquête exploratoire révèle l´indéniable existence du stress professionnel chez les salariés des entreprises (70,40% de ces salariés étaient en situation de job strain, 50,22% en iso strain selon le modèle de Karasek et 52,02% de ces salariés étaient en déséquilibre efforts/récompenses selon le modèle de Siegrist). Elle a permis également de montrer qu´au-delà des dispositions individuelles ou de la société à laquelle appartient le salarié, le milieu du travail joue un rôle prépondérant dans l´émergence du stress professionnel. Scientifiquement validés, les questionnaires de Karasek en complément de celui de Siegrist ont permis de retenir comme principaux facteurs de stress les grands efforts consentis à la réalisation du travail et le faible pouvoir de décision. Ces résultats traduisent le fait que le stress lié au travail chez les salariés dans les entreprises burkinabé est un vécu au quotidien dont les causes émanent de la charge de travail élevée et du faible pouvoir de décision. Cependant, ce vécu n´est pas exprimé. Ainsi, ces résultats ont mis en évidence le fait que l´expression du stress professionnel diffère d´une culture à une autre, car si dans les pays développés, le stress professionnel est reconnu et exprimé par les salariés, dans les pays en développement notamment le Burkina Faso, le stress n´est ni reconnu, ni exprimé.

### Etat des connaissances sur le sujet

- Le stress professionnel a été mesuré dans le domaine des entreprises privées burkinabé;
- Les facteurs de stress sont connus : gros effort consenti à la réalisation du travail et faible pouvoir de décision.

### Contribution de notre étude à la connaissance

- Le stress professionnel est vécu au quotidien par les salariés des EPF mais n´est pas exprimé.

## Remerciements

Nos remerciements sont adressés à toute l´équipe de recherche, aux chefs d´entreprises et aux salariés ayant acceptés de se prêter à nos enquêtes.
